# Multicenter comparison of three intraoperative hemoglobin trend monitoring methods

**DOI:** 10.1007/s10877-019-00428-3

**Published:** 2019-12-03

**Authors:** Richard L. Applegate II, Patricia M. Applegate, Maxime Cannesson, Prith Peiris, Beth L. Ladlie, Klaus Torp

**Affiliations:** 1 Department of Anesthesiology, Loma Linda University, Loma Linda, CA USA; 2 Present Address: Department of Anesthesiology and Pain Medicine, University of California Davis, Sacramento, CA USA; 3 Department of Cardiology, University of California, Davis, Sacramento, CA USA; 4 Department of Anesthesiology and Perioperative Care, University of California Irvine, Irvine, CA USA; 5 Present Address: Department of Anesthesiology and Perioperative Care, University of California Los Angeles, Los Angeles, CA USA; 6 Department of Anesthesiology, Mayo Clinic in Florida, Jacksonville, FL USA

**Keywords:** Hemoglobin, Point of care tests, Noninvasive measurement, Intraoperative monitoring

## Abstract

Transfusion decisions are guided by clinical factors and measured hemoglobin (Hb). Time required for blood sampling and analysis may cause Hb measurement to lag clinical conditions, thus continuous intraoperative Hb trend monitoring may provide useful information. This multicenter study was designed to compare three methods of determining intraoperative Hb changes (trend accuracy) to laboratory determined Hb changes. Adult surgical patients with planned arterial catheterization were studied. With each blood gas analysis performed, pulse cooximetry hemoglobin (SpHb) was recorded, and arterial blood Hb was measured by hematology (tHb), arterial blood gas cooximetry (ABGHb), and point of care (aHQHb) analyzers. Hb change was calculated and trend accuracy assessed by modified Bland–Altman analysis. Secondary measures included Hb measurement change direction agreement. Trend accuracy mean bias (95% limits of agreement; g/dl) for SpHb was 0.10 (− 1.14 to 1.35); for ABGHb was − 0.02 (− 1.06 to 1.02); and for aHQHb was 0.003 (− 0.95 to 0.95). Changes more than ± 0.5 g/dl agreed with tHb changes more than ± 0.25 g/dl in 94.2% (88.9–97.0%) SpHb changes, 98.9% (96.1–99.7%) ABGHb changes and 99.0% (96.4–99.7%) aHQHb changes. Sequential changes in SpHb, ABGHb and aHQHb exceeding ± 0.5 g/dl have similar agreement to the direction but not necessarily the magnitude of sequential tHb change. While Hb blood tests should continue to be used to inform transfusion decisions, intraoperative continuous noninvasive SpHb decreases more than − 0.5 g/dl could be a good indicator of the need to measure tHb.

## Introduction

Hemoglobin (Hb) measurement informs patient-specific perioperative transfusion decisions within the context of symptoms, comorbid conditions, surgical procedure, observed bleeding and hemodynamic performance [1, 2]. Hb measurement is a key component in many parts of patient blood management bundles [3], and is recommended between transfused red blood cell units if patient stability allows [4].

Hb measurement may be performed using clinical laboratory hematology (tHb; closest to the cyanmethemoglobin standard [5]), arterial blood gas cooximetry (ABGHb), or point of care Hb analyzers. However, the time needed for blood sampling and analysis can cause Hb measurement to lag clinical situations. In surgical settings in which blood loss may not be apparent or be difficult to estimate, continuous rather than intermittent Hb monitoring could provide earlier warning of decreasing Hb.

Multiwave pulse cooximetry noninvasively determines total hemoglobin (SpHb). SpHb has been tested in volunteers [6] and in a wide range of clinical settings [7]. Prior reports indicated wide limits of agreement between SpHb and tHb that suggest caution when using SpHb alone to guide transfusion decisions [7, 8]. However, it is possible that SpHb changes could provide useful information if the direction of SpHb change accurately reflects the direction of tHb change (trend). We defined trend accuracy as agreement of sequential changes in SpHb, ABGHb, or point of care Hb with sequential tHb changes. The aim of this multicenter study was to evaluate trend accuracy of three monitoring methods in patients undergoing surgery.

## Methods and materials

This collaborative prospective convenience sample observational study received Institutional Review Board approval at each of three USA academic medical centers prior to study initiation: Loma Linda University (LLU), Loma Linda, CA; Mayo Clinic in Florida (MCF), Jacksonville, FL; and University of California Irvine (UCI), Irvine, CA. Patient consent was obtained according to local IRB determination at each site. All study procedures were performed in accordance with the ethical standards of the institutional and research committees and with the 1964 Helsinki declaration and its later amendments or comparable ethical standards. This manuscript adheres to applicable STARD guidelines.

Adult patients were eligible for study participation if scheduled to undergo non-cardiac surgery in which arterial catheterization and expected repeated intraoperative blood gas analyzes were planned as part of patient care. Patients were excluded if pregnant or for skin abnormalities at the planned application site that would interfere with pulse oximetry (burns, scar tissue, nail polish, or infection). Attending anesthesiologists had discretion over anesthesia management, fluid administration and transfusion decisions, which were based on clinical settings (surgical considerations, ongoing bleeding, hemodynamic condition and/or any patient comorbid conditions) and institutional guidelines, which were in keeping with published guidelines [9, 10].

Multiwave disposable pulse cooximetry finger sensors (R125, Radical-7, Revision K, Masimo, Irvine, CA) were placed on the ring finger on the side of arterial cannulation with data continuously collected to computer. Oximeters were set to arterial mode to align with the source of blood samples. The SpHb algorithm continuously evaluates up to 6 min of data to calculate the displayed value. Whenever arterial blood gas analysis was performed, SpHb displayed at the time arterial blood was drawn was recorded, and arterial blood samples were obtained in appropriate collection tubes. Blood sample analysis was completed within 10 min. Each blood sample was analyzed twice using the same analyzer for tHb, ABGHb and aHQHb using:

- *Clinical Laboratory hematology analyzer Hb* (*tHb*; LLU—Sysmex XE5000, Sysmex America Inc., Lincolnshire, IL, USA; MCF—Sysmex XE5000, Sysmex America Inc., Lincolnshire, IL, USA or Coulter AcTdiff, Beckman Coulter, Indianapolis, IN, USA. UCI—Coulter LH 750 Hematology Analyzer, Beckman Coulter, Brea, CA, USA)
- *Arterial blood gas cooximetry Hb* (*ABGHb*; LLU—Radiometer ABL800; Radiometer, Copenhagen, Denmark. MCF—CCX or PhOX, Nova Biomedical, Waltham, MA, USA; UCI—Siemens RAPIDLab 1265, Siemens Healthcare Diagnostics, NY, USA)
- *Point of care Hb using arterial blood* (*aHQHb*; not capillary or finger stick samples; HemoCue HB 301, HemoCue America, Brea, CA, USA)

### Statistical methods

The primary outcome measure was trend accuracy using modified Bland–Altman analysis of difference between changes in SpHb, ABGHb or aHQHb and tHb changes to tHb changes as the measurement standard, to obtain bias and 95% limits of agreement between trends in SpHb, ABGHb, or aHQHb and tHb trend. Trends were defined as sequential change in Hb using results of the first analysis performed on each blood sample: Hb sample 2—Hb sample 1; Hb sample 3—Hb sample 2; etc. We included in analysis only samples for which all measures were available for trend calculations and did not exclude or separately analyze SpHb in low perfusion states.

Power and sample size calculation: A prior study of patients undergoing similar procedures [11] reported a median of 4 (up to 9) blood samples allowing an average of 3 trend calculations per patient. The mean bias of SpHb to tHb reported in prior studies in which the intended hematology analyzers were used [7] ranged from − 0.53 to 1.22, with standard deviation averaging 1.055. Using that standard deviation, setting alpha to 0.05 and power to 0.8, a sample size of 135 patients would be needed to estimate the precision of the 95% confidence interval of the difference among the 3 methods within 0.2 g/dl This is a conservative estimate of our true precision because patients would have repeated blood draws. Power analyses were performed using SAS 9.4 (SAS Institute, Cary, North Carolina, USA); statistical analyses were performed using JMP Pro version 13.2.0 (SAS Institute, Cary, North Carolina, USA) and Prism 8.1.0 (GraphPad Software, San Diego, California, USA).

## Secondary outcome measures

### Hb accuracy

Calculated Hb change could be impacted by Hb measurement accuracy. Accuracy of SpHb, ABGHb or aHQHb compared to tHb was evaluated by Bland–Altman analysis to determine bias and 95% limits of agreement. Hb measurement results were compared to clinically acceptable error as previously recommended [12].

### Hb measurement repeatability

Results of the 2 analyses performed on each blood sample were compared by Bland–Altman analysis to find bias and 95% limits of agreement for tHb, ABGHb and aHQHb.

### Agreement of change direction

The 95% limits of agreement obtained from Hb measurement repeatability analysis were used to define exclusion zones for change direction agreement analysis. Change direction agreement was assessed overall and for samples with tHb < 9.0 g/dl. This was reported as % agreement; 95% confidence interval.

## Results

Each center enrolled independently with one-hundred thirty-five patients studied (Loma Linda University n = 51; Mayo Clinic in Florida n = 58; University of California Irvine n = 26). On average patients had 4 samples obtained, ranging from 2 to 13. A total of 568 blood gas samples were drawn from these patients. Of these, 5 (0.88%) samples were missing a blood gas analysis result, 5 (0.88%) were missing SpHb and 7 (1.23%) had automated data collection errors, leaving 551 samples in which all measurements were available providing 416 changes to calculate trends for comparisons. Patient and procedure characteristics are shown in Table 1.

**Table 1:** Patient characteristics

Patient characteristics	All N = 135	LLU N = 50	MCF N = 59	UCI N = 26
Sex # (%)				
Female	70 (51.9%)	29 (58.0%)	26 (44.1%)	15 (51.9%)
Male	65 (48.1%)	21 (42.0%)	33 (55.9%)	11 (42.3%)
Age years	61 [50–69]	58.5 [44.5–68.5]	63 [54–70]	58.5 [47.8–68.3]
Weight kg	79.3 [66.1–96.2]	79.6 [64.9–98.9]	79.3 [66.6–92.6]	76.9 [64.6–96.4]
Body mass index kg m^−2^	28.0 [24.0–32.7]	28.0 [24.5–35.0]	28.1 [23.8–29.9]	27.9 [22.7–33.2]
Number of samples per patient	4	4	4	4
Range	[3–5] 2–13	[3–5] 2–6	[2–6] 2–13	[4–4] 2–4
1st intraoperative hemoglobin g/dl Range	10.8 [9.1–11.9] 5.9–14.7	10.8 [9.3–11.9] 5.9–14.7	10.1 [8.6–11.7] 6.8–13.8	11.7^a^ [10.3–12.9] 9.1–13.8
Intraoperative hemoglobin g/dl Range	10.1 [8.9–11.5] 4.9–14.7	10.0 [8.7–11.3] 4.9–14.7	9.5 [8.6–11.0] 5.9–14.1	11.5 [10.3–12.5] 8.4–14.3^b^
Sequential change in laboratory hemoglobin g/dl Range	− 0.1 [− 0.6 to 0.4] − 5.0 to 3.7	0.3 [− 0.8 to 0.3] − 5.0 to 3.2^d^	0 [− 0.7 to 0.5] − 3.3 to 3.7	0 [− 0.2 to 0.3] − 3.3 to 0.9
Duration of monitoring minutes	302 [181–390]	335 [257–386]	354 [266–437]	88^c^ [79–101]
Surgical procedure type #				
Major abdominal	58	19	26	13
Liver resection or transplant	25	3	17	5
Major orthopedic	16	8	5	23
Major urologic	16	3	11	2
Major gynecologic	12	11	0	1
Major vascular	6	6	0	0
Major neurosurgical	2	0	0	2

Characteristics of patients undergoing surgery with arterial catheterization at one of 3 academic medical centers: *LLU* Loma Linda University; *MCF* Mayo Clinic in Florida; *UCI* University of California Irvine. Results are median [25th to 75th percentile] except sex and procedure type which are number (%)

^a^First intraoperative hemoglobin higher at UCI than LLU (Hodges Lehman difference 0.9; 0.1–0.8 g/dl p = 0.03) and MCF (Hodges Lehman difference 1.3; 0.5–2.2 g/dl p = 0.003)

^b^Intraoperative hemoglobin higher at UCI than LLU (Hodges Lehman difference 1.4; 1.0–1.8 g/dl) and MCF (Hodges Lehman difference 1.7; 1.3–2.1 g/dl) both p < 0.0001

^c^Duration of monitoring less at UCI than LLU (Hodges Lehman difference − 237; − 268 to − 201 min) and MCF (Hodges Lehman difference − 264; − 298 to − 225 min) both p < 0.0001

^d^Sequential change more negative at LLU than MCF (Hodges Lehman L difference − 0.2; − 0.4 to 0 g/dl; p = 0.04) and UCI (Hodges Lehman difference − 0.3; − 0.4 to − 0.1 g/dl; p = 0.006)

### Trend accuracy

**Fig. 1:**
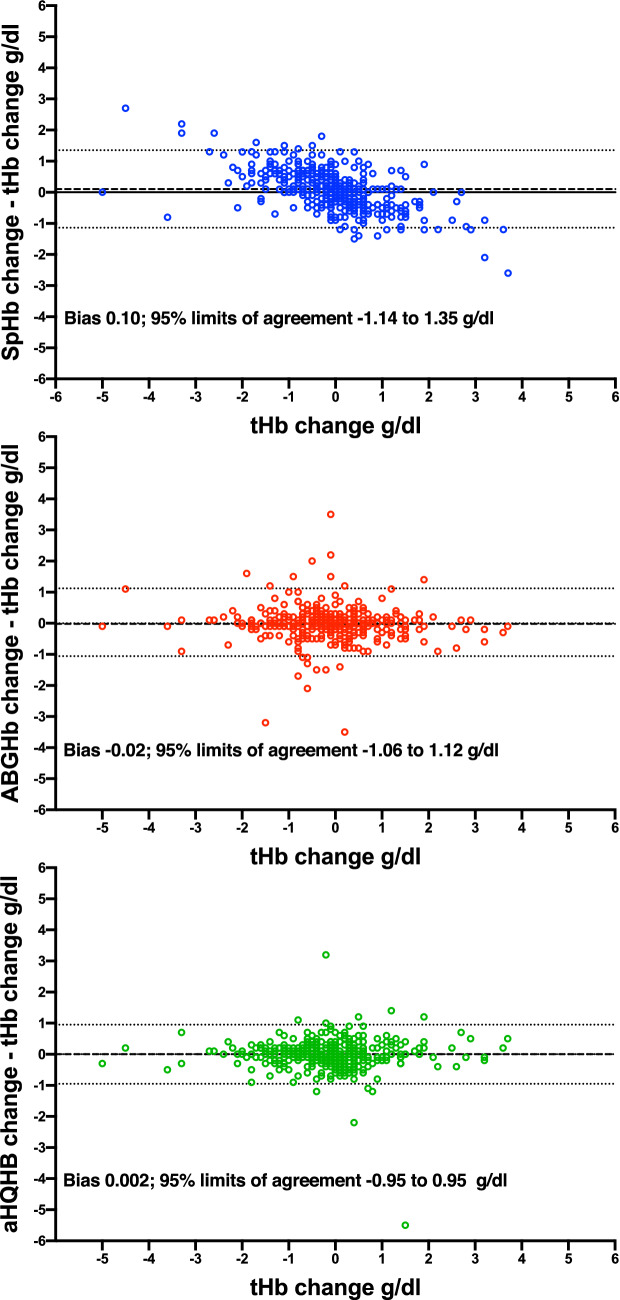
Modified Bland–Altman analysis of trend accuracy comparing 416 sequential changes in laboratory hematology analyzer hemoglobin (tHb) to the difference between tHb changes and paired sequential changes in top panel: pulse cooximetry hemoglobin (SpHb); middle panel: arterial blood gas cooximetry hemoglobin (ABGHb) and bottom panel: Hemocue point of care hemoglobin using arterial blood (aHQHb). Horizontal dotted lines indicate 95% limits of agreement (± 1.96 SD)

Modified Bland–Altman analysis showed small bias with slightly wider limits of agreement for SpHb trends compared to ABGHb or aHQHb trends (Fig. 1). Mean bias (limits of agreement g/dl) for SpHb was 0.10 (− 1.14 to 1.35); for ABGHb was − 0.02 (− 1.06 to 1.02); and for aHQHb was 0.003 (− 0.95 to 0.95).

### Hb accuracy

**Fig. 2:**
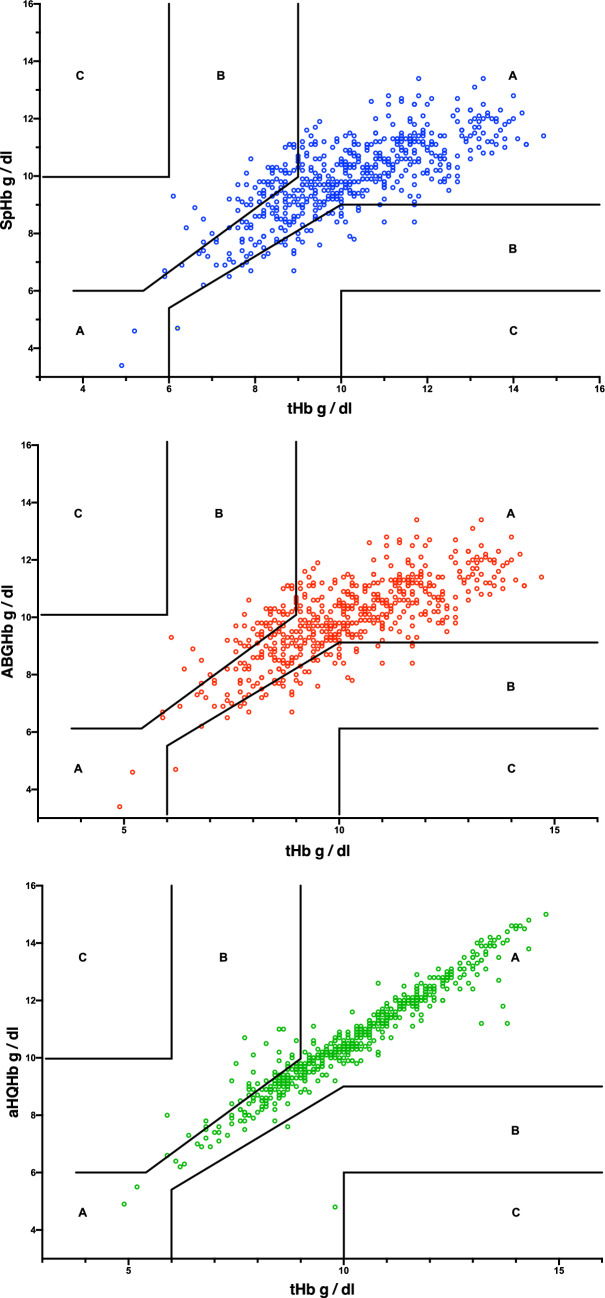
Clinical acceptability plot of absolute accuracy comparing hemoglobin determined by laboratory hematology analyzer (tHb) to hemoglobin determined by top panel: pulse cooximetry (SpHb); middle panel: arterial blood gas cooximetry (ABGHb) and bottom panel: Hemocue point of care using arterial blood (aHQHb). Compared to results for tHb: zone A indicates results within a clinically acceptable range (± 10%) at lower tHb; zone B indicates results that could represent a clinically significant error; and zone C indicates a potentially dangerous error in results from SpHb, ABGHb or aHQHb [12, 13]

Compared to tHb, mean bias (95% limits of agreement) for SpHb was 0.24 (− 2.05 to 2.53) g/dl; for ABGHb was − 0.36 (− 1.47 to 0.78) g/dl; and for aHQHb was − 0.43 (− 1.46 to 0.60) g/dl. Hb accuracy is plotted against clinically acceptable error [12, 13] in Fig. 2. None of the results were in the zone that has been proposed to potentially expose patients to larger risks [13].

### Measurement repeatability

Bias (95% limits of agreement) of results from the 2 analyses of each blood sample for tHb was 0.0055 (− 0.25 to 0.26) g/dl; for ABGHb was − 0.032 (− 0.61 to 0.54) g/dl; and for aHQHb was − 0.0004 (− 0.62 to 0.61) g/dl. These limits of agreement were used to set exclusion zones for comparing change direction agreement. A tHb change more than ± 0.25 g/dl was considered an increase or decrease, while a change more than ± 0.5 g/dl was considered an increase or decrease for SpHb, ABGHb and aHQHb.

### Agreement in change direction

**Fig. 3:**
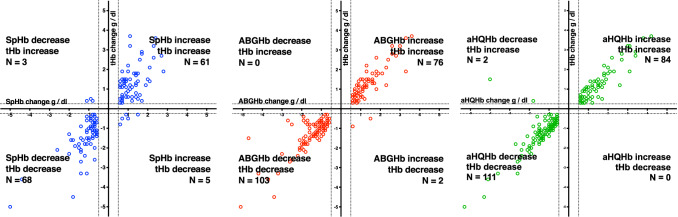
Four quadrant plots of changes in laboratory hemoglobin (tHb) compared to changes in hemoglobin determined by left panel: pulse cooximetry (SpHb); middle panel: arterial blood gas cooximetry (ABGHb) and right panel: point of care device using arterial blood (aHQHb). Dotted lines show limits of exclusion zones of ± 0.5 g/dl for SpHb, ABGHb and aHQHb and ± 0.25 g/dl for tHb based on repeated analysis of blood samples

**Fig. 4:**
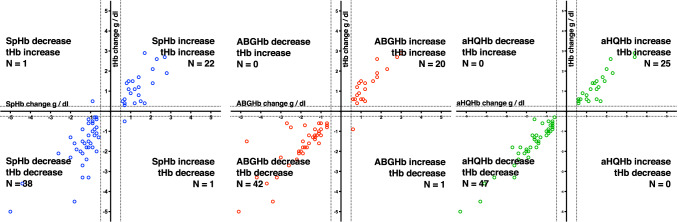
Four quadrant plots of changes in laboratory hemoglobin (tHb) when tHb < 9.0 g/dl compared to changes in hemoglobin determined by left panel: pulse cooximetry (SpHb); middle panel: arterial blood gas cooximetry (ABGHb) and right panel: point of care device using arterial blood (aHQHb). Dotted lines show limits of exclusion zones of ± 0.5 g/dl for SpHb, ABGHb and aHQHb and ± 0.25 g/dl for tHb based on repeated analysis of blood samples

Using the defined exclusion zones, change direction agreed in 129 of 137 SpHb changes (94.2%; 88.9–97.0%); 179 of 181 ABGHb changes (98.9%; 96.1–99.7%); and in 195 of 197 aHQHb changes (99.0%; 96.4–99.7%). Table 2 details change direction agreement for increases and decreases. Four quadrant plots using the defined exclusion zones are shown in Fig. 3. In all samples when SpHb, ABGHb or aHQHb increased but tHb decreased the decreases in tHb were smaller than − 1 g/dl. For tHb < 9.0 g/dl changes in SpHb, ABGHb or aHQHb more than ± 0.5 g/dl agreed with tHb change direction in all but 3 instances (Fig. 4). Most samples in which SpHb, ABGHb or aHQHb change was not more than ± 0.5 g/dl were associated with tHb changes within ± 0.25 g/dl. However, when tHb was < 9.0 g/dl, one ABGHb and 3 SpHb changes not more than ± 0.5 g/dl had an associated tHb decrease more than − 1 g/dl.

**Table 2:** Agreement of trend direction between tHb change more than ± 0.25 g/dl and SpHb, ABGHb and aHQHb change more than ± 0.5 g/dl

	Same direction as tHb trend N (%; 95% CI)	Not the same direction as tHb trend N (%; 95% CI)
Increase		
SpHb	61 (92.4%; 83.5–96.7%)	5 (7.6%; 3.3–16.5%)
ABGHb	76 (97.4%; 91.1–99.3%)	2 (2.6%; 0.7–8.9%)
aHQHb	84 (100%; 95.6–100%)	0
Decrease		
SpHb	68 (97.8%; 88.3–98.6%)	3 (4.2%; 1.4–11.7%)
ABGHb	103 (100%; 96.4–100%)	0
aHQHb	111 (98.2%; 93.8–99.5%)	2 (1.8%; 0.5–6.2%)

## Discussion

Intraoperative Hb trend accuracy limits of agreement were approximately ± 1 g/dl for ABGHb and aHQHb and slightly larger for SpHb trends. Considering the 95% confidence intervals for agreement in change direction overlapped, our findings suggest that changes more than ± 0.5 g/dl in SpHb, ABGHb and aHQHb provide similar information regarding the direction of intraoperative tHb change, but not necessarily the magnitude of that change. The impact of trend accuracy on transfusion decision-making is more significant when tHb is less than 9.0 g/dl as current guidelines suggest that red blood cell transfusion may be reasonable [14] for tHb < 8.0 g/dl, but usually unnecessary [9] for tHb > 10.0 g/dl. Analysis of change direction agreement suggests that if continuous SpHb is < 9 g/dl then a decrease more than − 0.5 g/dl could be a good indication to obtain a blood sample for tHb measurement.

Repeatability analysis demonstrated limits of agreement that have implications for Hb accuracy and trend monitoring studies. The exclusion zones we defined based on 95% limits of agreement for ABGHb and aHQHB suggest that in our patients, a change up to ± 0.5 g/dl may not reflect a real change in circulating hemoglobin measured by tHb. Similarly, reported tHb changes of ± 0.25 g/dl may not represent real changes in circulating Hb. Our findings support use of an exclusion zone when comparing Hb analyzer trend performance and highlight the need to provide specific education regarding the exclusion zone appropriate to clinical use of any Hb monitor. This exclusion zone could be different in other centers or when using varying analyzers.

The SpHb results can be compared to prior reports of single center studies. A study of volunteers found 95.4% SpHb change agreement in 22 samples with tHb < 10.0 g/dl [15], which is similar to what we found. Using venous blood samples, a study of 70 trauma patients reported similar bias with narrower limits of agreement (− 0.05; − 0.62 to 0.51 g/dl) of SpHb change to tHb change than we report [16]. The number of patients and change calculations is smaller than included herein and reasons for the narrower limits of agreement are not clear, but may be partially explained by the multicenter source of our data. Studies of 48 vascular surgery patients [17] and of 70 patients undergoing major orthopedic surgery [18] concluded SpHb had clinically acceptable trending with ABGHb or tHb. In 49 patients undergoing spine surgery, change concordance was 85.1% when excluding SpHb with perfusion index under 1 [19]. When using the exclusion zones determined by measurement repeatability analysis but not excluding SpHb based on low perfusion index, SpHb concordance was 94.2%. Our findings are also better than reported in 69 patients undergoing spine or cancer surgery that employed a 1 g/dl exclusion zone [20]. A study of patients undergoing abdominal or pelvic surgery found 41 of 269 changes at any Hb had increased SpHb when ABGHb decreased, including 14 in which the decrease was more than − 1 g/dl [11]. Our finding of better trend direction agreement may be related to use of a newer SpHb version in the present study. We also found that Hb accuracy is improved compared to reports using earlier versions of the SpHb monitor [6–8, 11].

Study limitations include having only about 10% of change samples with tHb ≤ 8.0 g/dl (43 of 416), which impacts our ability to assess clinical utility at very low tHb. However, change direction agreement was good for tHb < 9.0 g/dl. The number of samples in which SpHb, ABGHb or aHQHb change were not more than ± 0.5 g/dl represents a potentially problematic grey zone for assessing Hb change. Although at tHb < 9.0 g/dl only 1 ABGHb and 3 SpHb decreases in this grey zone had associated tHb changes more than − 1.0 g/dl, potential clinical impact of these is not clear and could be evaluated in a future prospective study. We studied SpHb Rev K but newer SpHb versions could potentially have different or better trend accuracy. This could be assessed in a future study. The use of arterial blood can be seen as problematic for generalization to clinical settings in which venous or capillary blood is more commonly sampled. We used arterial blood samples as we studied patients at risk for blood loss and routinely use arterial catheters in such patients to facilitate care and blood sampling. Although venous blood may be easier to obtain in some clinical situations, setting SpHb monitors to arterial mode and using only arterial blood removed potential confounding that a mix of arterial and venous blood samples could have introduced into trend accuracy analyses. Differences between arterial and venous Hb have been reported as 0.2–0.3 g/dl [21–23]. We would expect trend calculations to be similar to our findings if only venous blood samples were tested. Use of arterial blood samples to determine aHQHb likely provides different results compared to capillary finger stick blood samples. However, our patients had as many as 13 samples which would have required many finger or ear lobe punctures to allow comparison to capillary samples, thus we chose to use arterial blood samples. Compared to our results, wider limits of agreement between tHb and point of care Hb have been reported when using capillary finger or toe stick samples in patients undergoing cesarean section [24], with gastrointestinal bleeding [25], or in the emergency room [26] and correlated poorly to arterial and venous tHb in intensive care patients [27]. Finally, we did not ascertain whether blood loss was suspected by clinical conditions but the range of tHb change in sequential samples was − 5.0 to + 3.7 g/dl, which reflects blood loss as well as transfusion decisions made by the anesthesiologists caring for the patients.

Hb measurement is essential to making patient centered transfusion decisions [28] which should improve outcomes [29] and reduce transfusion requirements [30–32]. Hb measurement is particularly important when blood loss is not obvious or is difficult to estimate during surgery as blood sampling to determine tHb can lag clinical situations. Surgical patients are reported to receive both unnecessary or excessive transfusion [33, 34] and anemia and transfusion can increase perioperative morbidity and mortality [35–37] for most but not all patients [38, 39]. However, postoperative outcome is reportedly better using less restrictive transfusion practices following some types of surgery [40], in elderly patients [41, 42] or in patients with cardiovascular disease [43, 44].

### Conclusions

We found that SpHb, ABGHb and aHQHb changes more than ± 0.5 g/dl have similar correlation to the direction but not necessarily the magnitude of tHb change during surgery. The similar agreement in trend direction suggests that clinicians can choose which to use based on availability or preference, although continuous SpHb monitoring may provide useful ongoing Hb trend information. Continuous noninvasive SpHb decreases exceeding − 0.5 g/dl may prompt a decision to obtain a confirmatory tHb measurement if low tHb is clinically suspected, but not replace blood Hb measurement in guiding transfusion decision making. Importantly, this study did not evaluate the transfusion impact of using these monitors so the transfusion impact of continuous noninvasive Hb monitoring needs to be studied prospectively.
